# Diabetic Retinopathy Prediction by Ensemble Learning Based on Biochemical and Physical Data

**DOI:** 10.3390/s21113663

**Published:** 2021-05-25

**Authors:** Zun Shen, Qingfeng Wu, Zhi Wang, Guoyi Chen, Bin Lin

**Affiliations:** 1 School of Informatics, Xiamen University, Xiamen 361005, China; shenz@stu.xmu.edu.cn (Z.S.); 24320172203111@stu.xmu.edu.cn (G.C.); libiloli@outlook.com (B.L.); 2 Department of Microelectronics and Nanoelectronics, Tsinghua University, Beijing 100876, China; wangzhi18@mails.tsinghua.edu.cn

**Keywords:** XGBoost feature selection, stacking ensemble learning, model fusion, diabetic retinopathy prediction

## Abstract

(1) Background: Diabetic retinopathy, one of the most serious complications of diabetes, is the primary cause of blindness in developed countries. Therefore, the prediction of diabetic retinopathy has a positive impact on its early detection and treatment. The prediction of diabetic retinopathy based on high-dimensional and small-sample-structured datasets (such as biochemical data and physical data) was the problem to be solved in this study. (2) Methods: This study proposed the XGB-Stacking model with the foundation of XGBoost and stacking. First, a wrapped feature selection algorithm, XGBIBS (Improved Backward Search Based on XGBoost), was used to reduce data feature redundancy and improve the effect of a single ensemble learning classifier. Second, in view of the slight limitation of a single classifier, a stacking model fusion method, Sel-Stacking (Select-Stacking), which keeps Label-Proba as the input matrix of meta-classifier and determines the optimal combination of learners by a global search, was used in the XGB-Stacking model. (3) Results: XGBIBS greatly improved the prediction accuracy and the feature reduction rate of a single classifier. Compared to a single classifier, the accuracy of the Sel-Stacking model was improved to varying degrees. Experiments proved that the prediction model of XGB-Stacking based on the XGBIBS algorithm and the Sel-Stacking method made effective predictions on diabetes retinopathy. (4) Conclusion: The XGB-Stacking prediction model of diabetic retinopathy based on biochemical and physical data had outstanding performance. This is highly significant to improve the screening efficiency of diabetes retinopathy and reduce the cost of diagnosis.

## 1. Introduction

Diabetes is one of the fastest growing health challenges in the 21st Century. According to the Global Diabetes Atlas [1], there will be 578 million adults with diabetes by 2030. Diabetic retinopathy is one of the most serious complications of diabetes, and it is the commonest cause of legal blindness in the working age population of developed countries [2], so the prevention of diabetic retinopathy cannot be ignored. With the spread of sensor devices and hospital information technology, diabetes data resources are becoming more available. Disease prediction methods based on data mining are increasingly used in real disease diagnosis scenarios, which can also significantly help the prediction and diagnosis of diabetic retinopathy.

Diabetic retinal data include image data and non-image structured data. According to different datasets in the diabetic retinopathy task, different methods are used in data mining. For massive retinal image data, experts often use deep learning to solve problems [3,4]. For structured data, the main prediction method is machine learning, for example: SVM, decision tree, and LR [5]; and the bagging ensemble classifier [6]. In the chronic diabetic retinopathy dataset, the medical association among diseases and physical examination indicators is more complex. For small high-dimensional samples with complex feature association, simple models and individual learners such as SVM and LR easily underfit and have poor performance.

Complex feature association affects the effect of data mining and has higher requirements for algorithm selection. Ensemble learning is a good choice. Ensemble learning produces a group of individual learners and then combines them with some strategies, such as bagging, boosting, and stacking [7]. It has been proven to be better than a single model, which is efficient to increase the accuracy and stability of classification algorithms [8]. However, the boosting and stacking methods are rarely used to predict diabetic retinopathy. For the ensemble algorithms, boosting is an effective and popular ensemble method in machine learning. Gradient Boosting Decision Trees (GBDTs) such as GBDT [9], XGBoost [10], LightGBM [11], and CatBoost [12] have become very successful in recent years, with many awards in machine learning and data mining competitions. In ensemble learning, stacking is a general ensemble method in which many base classifiers are combined using one meta-classifier, which learns from their outputs to reduce the limitations of a single model. Stacking has been proven to be an efficient model combination method that can improve the performance of a single model.

Therefore, this paper took the data of structured diabetic retinopathy as the research object and proposed new methods based on ensemble learning in feature selection and model construction. The contributions of this paper are as follows:

1. A new wrapped feature selection algorithm, XGBIBS (Improved Backward Search Based on XGBoost), was proposed to reduce feature redundancy and improve the effect of a single ensemble learning classifier. The buffer feature subset was added to make it possible to operate on multiple features, and XGBIBS searches for the optimal subset in the sorting space based on the different feature metrics of XGBoost;
2. A stacking model fusion method, Sel-Stacking (Select-Stacking), was proposed to improve the performance of a single model. There were two improvements to the algorithm. Sel-Stacking not only kept Label-Proba as the input matrix of the meta-classifier, but also determined the optimal combination of base classifiers by a global search;
3. A diabetic retinopathy prediction model, XGB-Stacking, was constructed to predict the risk of diabetic retinopathy by combining the XGBIBS feature selection algorithm and the Sel-Stacking model fusion method.

The remainder of the paper is organized as follows. Section 2 reviews related works, including the prediction of diabetic retinopathy, feature selection, and the stacking model fusion method. Section 3 focuses on the method to predict diabetic retinopathy and contains a brief introduction of the dataset, the XGBIBS feature selection algorithm, the Sel-Stacking model fusion method, and the evaluation matrix. The experimental setup and results are analyzed and discussed in Section 4. Finally, the conclusions are drawn in Section 5.

## 2. Related Work

### 2.1. Prediction of Diabetic Retinopathy

Experts deal with the prediction of diabetic retinopathy using various methods. Tsao et al. [5] built a prediction model for the DR in type 2 diabetes mellitus using data mining techniques including support vector machines, decision trees, artificial neural networks, and logistic regressions. The experiment showed that appropriate machine learning algorithms combined with discriminative clinical features could effectively detect diabetic retinopathy. Somasundaram et al. [6] designed the Machine Learning Bagging Ensemble Classifier (ML-BEC). Features of diabetic retinopathy disease diagnosis were initially extracted by applying t-distributed Stochastic Neighbor Embedding (t-SNE), and experiments suggested that ML-BEC could achieve better classification accuracy and was efficient for further reducing the diabetic retinopathy classification time. Ramani et al. [13] proposed a novel method that utilized retinal image analysis and data mining techniques to accurately categorize the retinal images as normal, diabetic retinopathy, and glaucoma-affected. The novel method included the Fisher ratio algorithm used in feature selection, as well as C4.5 and random forest, achieving the best classification accuracy.

### 2.2. Feature Selection Algorithm

#### 2.2.1. Wrapped Feature Selection Based on Heuristic Search

In different feature selection algorithms, the wrapped method tends to give superior performance compared with filters and embedded machine learning models [14]. At present, the wrapped feature selection algorithm based on heuristic search strategy is a research hotspot [15]. There are many different research works on wrapped feature selection algorithms based on heuristic search, such as random search and sequential search. Tan et al. [16] proposed a framework based on a Genetic Algorithm (GA) for feature subset selection that combined various existing feature selection methods. This approach could accommodate multiple feature selection criteria and find small subsets of features that performed well for a particular inductive learning algorithm to build the classifier. Sequence search is also a hot topic. Nakariyakul et al. [17] proposed a new Improved Forward Floating Selection (IFFS) algorithm. An additional search step called “replacing the weak feature” was added to check whether removing any feature in the currently selected feature subset and adding a new one at each sequential step could improve the current feature subset. Fallahpour et al. [18] proposed the Sequential Floating Forward Selection (SFFS) algorithm, and the SFFS-SVM ensemble classifier could be considered a promising addition to existent models when confronting the FDP issue. Guyon et al. [19] proposed a new method of gene selection utilizing support vector machine methods based on Recursive Feature Elimination (RFE). The genes selected by SVM-RFE yielded better classification performance and were biologically relevant to cancer. Li et al. [15] proposed a new wrapped feature selection algorithm, XGBSFS (XGBoost Sequential Floating Selection), and Improved Sequential Floating Forward Selection (ISFFS) was applied to search for the feature subset to achieve high quality.

Different wrapped feature selection methods based on the heuristic search strategy have defects. The time cost of random search is high. The current sequential search strategies are often based on sequential forward search and operate on a single feature in the search process, which easily leads to the appearance of redundant features or makes it difficult to consider the statistical correlation of multiple features.

#### 2.2.2. Feature Selection Algorithm Based on XGBoost

The XGBoost feature selection method has been used in different fields, and it has achieved good performance [15,20,21]. Li et al. [15] proposed a new feature selection method, XGBSFS. In XGBSFS, the thought process of building trees in XGBoost was used as a reference, and different feature importance metrics were measured to avoid the limitation of a single importance metric. Sang et al. [20] proposed feature selection based on XGBoost to improve the performance of DBP prediction effectively, and the XGBoost algorithm could provide better feature ranking than the random forest method. Chen et al. [21] employed XGBoost to reduce the feature noise and performed a dimensionality reduction through gradient boosting and average gain. The experiment obtained the top-ranked features based on the descending order of feature importance to characterize the PPIs.

XGBoost is often used in sequence forward search or the filtered method in feature selection. This method usually loses more feature information, which makes it difficult for XGBoost feature importance metrics to play a greater role.

### 2.3. Multi-Model by Stacking Ensemble Learning

The multi-model by stacking ensemble learning method has outstanding performance in many fields, and it also has good applicability in disease diagnosis tasks in the medical field [22,23,24,25]. Wang et al. [22] proposed a stacking-based ensemble learning method that simultaneously constructed the diagnostic model and extracted interpretable diagnostic rules. A random forest classifier-based stacking technique was explored for the integration of the base learners. Rawat et al. [23] explored the usage of stacking for two models, and the gradient boosting machine and artificial neural network were used in the prediction of dementia. The experimental results showed that the stacking model was better than the single model. Hammam et al. [24] proposed a stacking deep learning methodology to produce the best results of COVID-19 classification, which produced test accuracy of 98.6%. Ji et al. [25] proposed a classification strategy of multi-feature combination and the Stacking-DWKNN algorithm, which consisted of four modules. The average accuracy obtained was 99.01%.

Among the above stacking model fusion methods, most of them specify the combination of classifiers, and a few of them explore the combination of classifiers. In the stacking method, how to choose the combination of classifiers is also a question worth exploring.

## 3. Materials and Methods

### 3.1. Materials

The diabetic complication predicted by this study as diabetic retinopathy. This dataset was provided by the China National Clinical Medical Science Data Center. It is a publicly available database. This dataset is a structured dataset of diabetic retinopathy with numerical variables and qualitative variables, and it does not contain image data. The data monitored by different sensors included the biochemical indicators and physical indicators of diabetic patients, as well as the disease information of patients.

The original dataset was preprocessed before using it in the experimental method. The processing methods included deleting samples with overwhelming missing features, filling in missing values, and deleting outliers.

The processed experimental data included a total of 2990 samples, 68 features, and 1 label, among which the label indicated whether a sample contained diabetic retinopathy. A label value of zero indicated a diabetic patient with diabetic retinopathy, and a label value of one indicated a diabetic patient without diabetic retinopathy. There were 1496 diabetic patients with diabetic retinopathy and 1494 diabetic patients without diabetic retinopathy. All the features are shown in Table 1, including the basic patient information, patient disease information, and various biochemical indicators.

Before applying feature selection and model construction, the dataset was described statistically to understand its sample composition and value proportion. Table 2 shows the sex distribution of the samples, while Table 3 shows the age distribution of the samples. Table 4 shows the distribution of other basic qualitative information about the samples.

There were 1869 male and 1121 female samples, accounting for 62.51% and 37.49%, respectively. The age range covered from 19 to 93 years old, with the proportion of male samples being high. The cumulative proportion of 51–70-year-olds accounted for 62.81%, which means that most of the patients were elderly patients. As can be seen from Table 4, the majority of patients were Han ethnicity (the NATION value was 0), accounting for 95.65%, and the majority of patients were married (the MARITAL_STATUS value was 0), accounting for 97.89%.

### 3.2. XGBIBS Feature Selection

**Figure 1:**
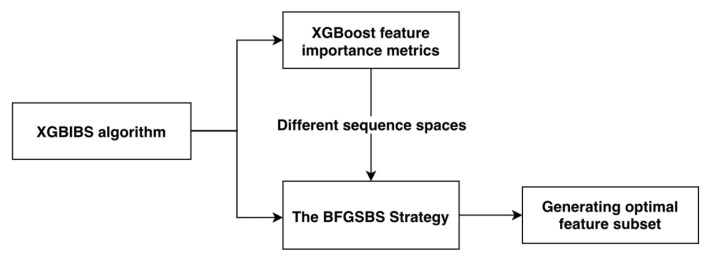
Two elements of the XGBIBS algorithm.

The XGBIBS feature selection algorithm includes two elements, which are shown in Figure 1. First, XGBoost provides different feature importance metrics to form two feature-ranking spaces for feature search. Second, Buffer Floating Generalized Sequential Backward Search (BFGSBS) is used to search for the optimal subset.

#### 3.2.1. XGBoost Feature Importance Metrics in the XGBIBS Algorithm

Feature importance metrics measure the importance of a feature in the construction of a model. The reason for choosing different feature importance metrics provided by XGBoost is to fully obtain the internal correlation among features and targets and improve the search efficiency. The feature importance score provided by XGBoost can represent the value of the feature in the model to enhance the construction of the decision tree, which is not a simple statistical linear relationship.

The base classifiers of the XGBoost algorithm support two choices: linear classifier and tree model. The importance metrics of XGBoost features in this paper were based on the tree model. In the process of building the tree model, XGBoost starts from the root node, and the feature is selected at each layer, which makes the tree obtain the maximum gain for segmentation. The importance of this feature increases when it is used to segment more times or the average gain of each feature segmentation becomes larger. In the process of the continuous segmentation of a tree, the calculation formula of the gain is as shown in Equation (1).

(1) $$Gain=\frac{1}{2}\left[\frac{G_{L}^{2}}{H_{L}+\lambda }+\frac{G_{R}^{2}}{H_{R}+\lambda }-\frac{{\left(G_{L}+G_{R}\right)}^{2}}{H_{L}+H_{R}+\lambda }\right]-\gamma$$

Common feature importance metrics in XGBoost are the gain, cover, weight, total_gain, and total_cover. These are shown in Table 5. Feature importance metrics can be obtained through the parameter in XGBoost “feature_importances_”, and different metrics of “feature_importances_” can be set to provide different metrics.

The weight is the number of times that a feature is used to split the data. Its calculation is shown in Formula (2), where *X* is the set of specified features classified into leaf nodes. The calculation formula of the gain is Formula (3), and the calculation formula of the gain is shown in Formula (1). The calculation formula of the cover is shown in Formula (4). The calculation formula of the total_gain is shown in Formula (5), while the calculation formula of the total_cover is shown in Formula (6).

(2) Weight=|X|

(3) $$gain=\frac{\sum Gain_{X}}{\left|X\right|}$$

(4) $$cover=\frac{\sum cover_{X}}{\left|X\right|}$$

(5) $$total\_gain=\sum Gain_{X}$$

(6) $$total\_cover=\sum cover_{X}$$

Before the XGBoost feature importance metrics are used in the search phase of the XGBIBS feature selection algorithm, the following processing is undertaken:

1. It filters out the features with zero importance. In tree segmentation, it is inevitable that features with a zero importance metric will appear. Most of the features with zero importance are not distinguishable from the samples, as their information value is very low. The XGBIBS feature selection algorithm can filter out the features with zero importance so that they cannot enter the feature search space;
2. It outputs multiple different feature importance metrics at the same time. It is necessary to construct two ranking spaces for the sequence search according to certain rules. After XGBoost calculates the feature importance metrics, it outputs a variety of rankings to be used in the XGBIBS feature selection algorithm. Different feature importance metrics of the BFGSBS strategy can be chosen arbitrarily.

#### 3.2.2. The BFGSBS Strategy in the XGBIBS Algorithm

The BFGSBS strategy in this study implemented floating generalized backward search by increasing the buffer subset. In addition, the BFGSBS strategy constructed two feature-ranking spaces through two importance metrics, $i_{1}$ and $i_{2}$, provided by XGBoost. The feature ranking queue for deleting feature subsets and that for adding features were different. The buffer subset of the BFGSBS strategy can fully consider the correlation among multiple features, and through two feature important metrics, it provides a richer combination of features and overcomes the limitations of a single metric.

The implementation steps of the BFGSBS strategy are as follows:

According to the XGBoost feature importance metric $i_{1}$, the features are sorted from small to large to generate the $I_{1}$ queue.

According to the other XGBoost feature importance metric $i_{2}$, the features are sorted in the opposite way in order to generate the $I_{2}$ queue.

A buffer feature subset is established starting with the full set of features *O*.

Stage 1 is the sequence backward deletion:

1. Delete the *N*th feature from the buffer feature subset *O* each time (the starting value of *N* is 1) according to the feature importance queue $I_{1}$, and the buffer feature subset *O* is updated;
2. Use the new buffer feature subset *O* to calculate the evaluation function. If the result is better than that of the optimal evaluation function, save this buffer feature subset as a new optimal feature subset Best_O;
3. After this round of operation, N=N+1, and go to Stage 2
  Stage 2 is the floating forward increase:
4. Search for a feature that is not in the buffer feature subset *O* and in turn from the feature importance queue $I_{2}$;
5. If this feature is added to the buffer feature subset *O*, the effect of the evaluation function is improved. Then, the buffer feature subset *O* is updated, and the buffer feature subset is saved as a new optimal feature subset, Best_O;
6. End this stage after traversing the order from beginning to end, and return to Stage 1.

After multiple iterations, an optimal feature subset with the least number of features and the highest evaluation function effect is finally obtained.

The two feature importance metrics of the BFGSBS strategy are provided by the XGBoost algorithm. The BFGSBS strategy and XGBoost feature metrics together constitute the XGBIBS feature selection algorithm.

**Figure 2:**
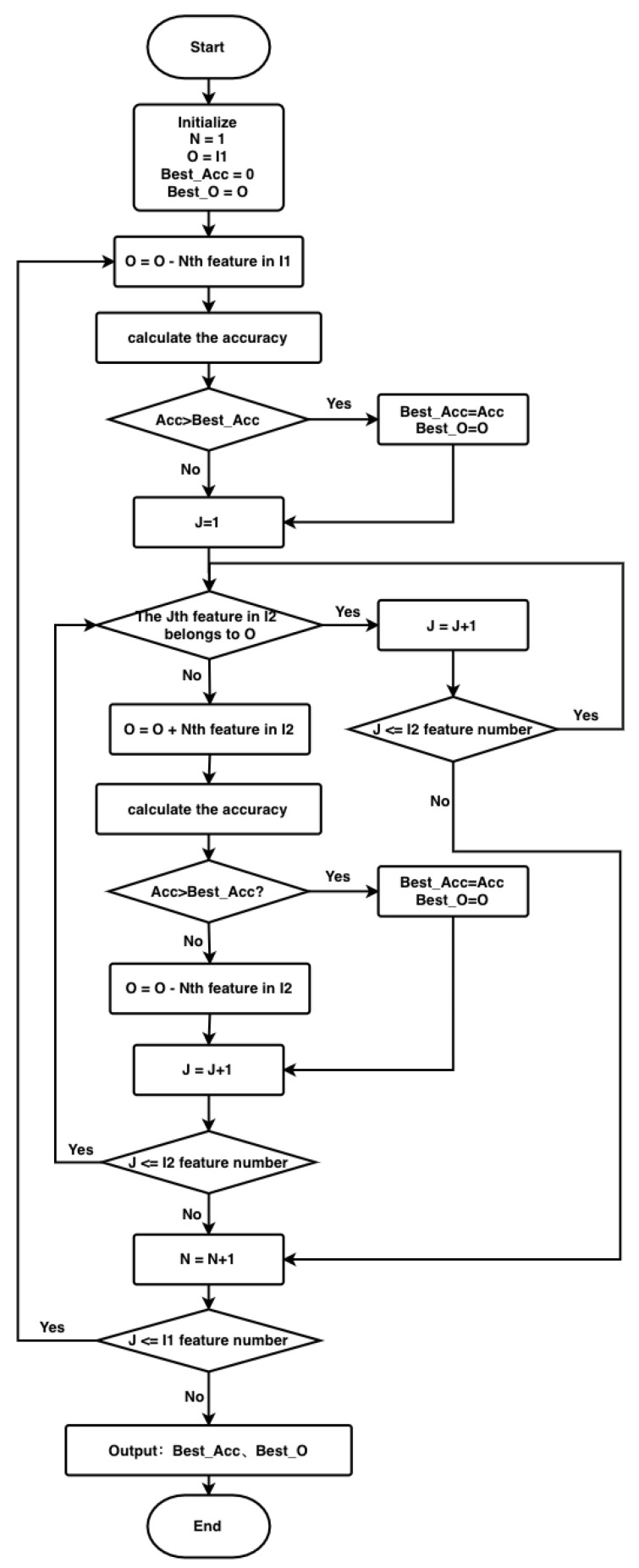
The flowchart of the XGBIBS algorithm (the BFGSBS strategy).

The flowchart of the XGBIBS algorithm (the BFGSBS strategy) is shown in Figure 2. The symbols in Table 6 are used when describing the algorithm. The pseudo-code of the XGBIBS algorithm is given in Algorithm 1.

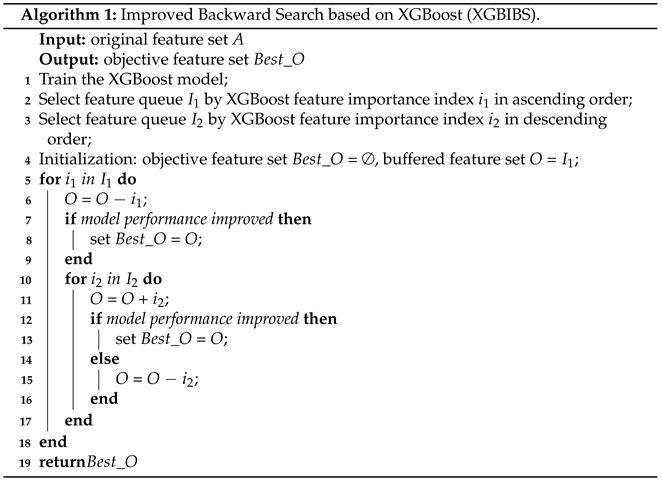



#### 3.2.3. Time Complexity of the XGBIBS Algorithm

This section analyzes the time complexity of the XGBIBS feature selection algorithm. The maximum depth of the tree constructed by XGBoost in the XGBIBS algorithm is *X*, and there are *K* trees in total. Assume that the training set has *N* samples and a total of *M* features, without missing features and samples. The number of non-zero importance features obtained by XGBoost feature importance ranking is *m*; for the XGBoost algorithm, the time complexity of generating all the feature presorting is O(mNlogN); since the results of global presorting can be reused in the later node splitting, no extra time to sort needs to be consumed. For the process of tree model construction, the time complexity of traversing the partition point of each layer is O(MN), so the time complexity of building K trees is O(MNKX). The average time complexity of the quick sort algorithm is O(mlogm). In BFGSBS, the time complexity of the sequence backward deletion process is O(m) because there are *m* features in the sequence $I_{1}$; the floating forward add procedure is also traversed from beginning to end on sequence $I_{2}$, the features if which are less than *m*, so the time complexity of the adding process is O(m); the time complexity of the base classifier is O(W). The total time complexity of BFGSBS is $O(m^{2}*W)$. Taking the k-Nearest Neighbor (KNN) as an example, the time complexity of the KNN classifier O(W) is O(mNlogN). Therefore, the time complexity of BFGSBS search is $O(m^{3}NlogN)$. Therefore, the total time complexity of the XGBIBS algorithm is shown as in Formula (7).

(7) $$O\left(XGBIBS\right)=O\left(mNlogN+mlogm+MNKX+m^{3}NlogN\right)$$

### 3.3. Multi-Model by Sel-Stacking Ensemble Learning

**Figure 3:**
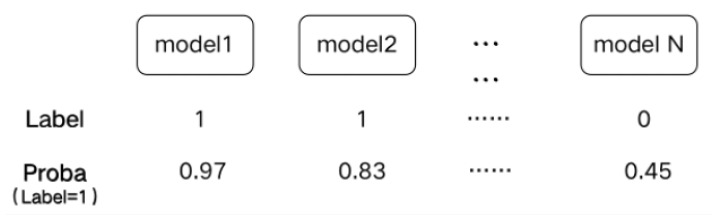
Label-Proba matrix of a sample.

Considering the limited number of base learners and the small amount of data in this experiment and in order to prevent the problems of overfitting and local optimization of the model combination, this study made the following improvements to the traditional stacking model fusion algorithm:

1. The Sel-Stacking method changes the input of meta-classifiers. In order to avoid overfitting, the output label and Proba of the base classifiers are retained and used as the input of the meta-classifier at the same time. This prediction is a binary classification problem. A single learner outputs the predicted label and the corresponding classification probability Proba. The Label-Proba matrix predicted by one base classifier of one input sample is shown in Figure 3. For each sample, N base classifiers produce a 2*N output matrix, and M samples with N base classifiers produce a 2M*N Label-Proba matrix.
2. The Sel-Stacking method improves the combination of learners and selects the optimal combination based on the data. For the model of the base classifiers, a variety of classifiers that have applied the XGBIBS feature selection algorithm were respectively connected, and the model was trained with six-fold cross-validation. The Sel-Stacking method adds a feature selection process between the base classifiers and the meta-classifier through a global search to select the best set of base classifiers.

The pseudo-code of the Sel-Stacking algorithm is summarized in Algorithm 2.

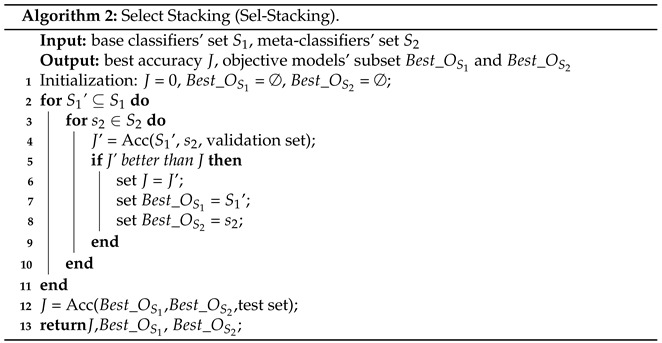



Acc($S_{1}$,$S_{2}$, dataset X) outputs the accuracy of meta-classifier $S_{2}$, whose input is the prediction matrix generated by base classifiers’ set $S_{1}$ on dataset X.

The computational complexity of the Sel-Stacking model fusion algorithm can be divided into two parts. When we use *M* base learners to fit a dataset with *N* rows of data, the first part is *K*-fold stacking, the time complexity of which is $O(K*\sum_{m=1}^{M}O_{m})$; the time complexity of the base classifiers *m* is $O_{m}$. The second part trains the beta learner SVM with the dataset generated by the *M* base classifiers. Since we used a global search to find the best combination of base classifiers, its time complexity is $O(2^{M}*O_{SVM})$. Thus, the whole time complexity of the Sel-Stacking is shown as in Formula (8).

(8) $$O\left(Sel-Stacking\right)=O\left(K*\sum_{m=1}^{M}O_{m}+2^{M}*O_{SVM}\right)$$

### 3.4. XGB-Stacking Model Based on the XGBIBS Algorithm and the Sel-Stacking Method

The method for predicting diabetic retinopathy in this study is called the XGB-Stacking model, which was divided into two steps: XGBIBS feature selection and the Sel-Stacking multi-model fusion process. Feature selection on all classifiers was first performed by the XGBIBS algorithm. All the classifiers were used as the optional base classifiers for model fusion.

**Figure 4:**
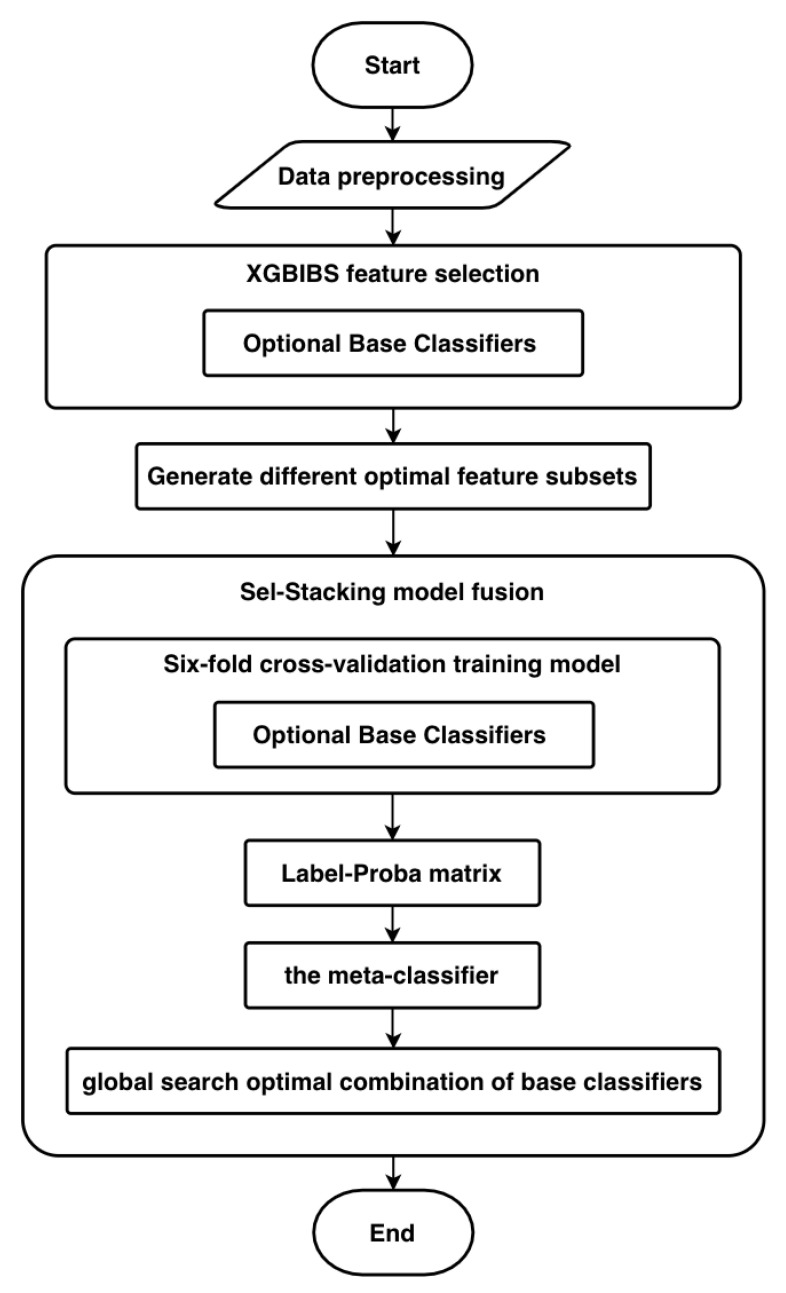
The flowchart of the XGB-Stacking model.

The flowchart of the method XGB-Stacking is shown in Figure 4.

### 3.5. Performance Evaluation Matrix

Generally, feature selection had two evaluation indicators in the classification problem experiment: classification accuracy and feature dimension reduction.

Classification accuracy (Acc) is defined as the proportion of the number of correctly classified samples to the overall number of samples, which is shown in Formula (9). NCC represents the Correct Number of Classifications, while NAS represents the total instances of the dataset. Feature Dimensionality Reduction (DR) refers to the ratio of the number of unused features to the number of original features, which is shown in Formula (10), where NSF represents the Number of Selected Features and that of all features. In the model fusion, only the accuracy was used as the evaluation matrix.

(9) $$Acc=\frac{NCC}{NAS}$$

(10) $$DR=1-\frac{NSF}{NAF}$$

## 4. Experiments and Results Discussion

Because there were two methods proposed, the XGBIBS feature selection algorithm and the Sel-Stacking model fusion method, this study performed experiments to verify the effectiveness of the feature selection method and the model fusion method, respectively.

### 4.1. Experimental Setup

#### 4.1.1. Experimental Environment

All experiments on the dataset of diabetic retinopathy were implemented on a client of the data provider on a Dell PowerEdge T640 workstation, running Windows 7, with Genuine Intel(R)2.60 GHz CPUs; all codes were implemented with Python 3.6.

#### 4.1.2. Dataset Partition

The dataset was divided into a training set used to train the model, a validation set to prevent overfitting, and an independent test set to test the generalization ability and prediction effect of the model. The dataset partition ratio was 6:2:2. The selection of the dataset segmentation ratio was based on the small sample size of this dataset, with a total of 2990 samples. The proportion of the training set should be slightly higher to ensure the effectiveness of model training, and the proportion of the verification set and test set should not be too small, so as to ensure that the generalization ability of the model is convincing. There were 1794 samples for the training set, 598 samples for the validation set, and 598 samples for the independent test set.

#### 4.1.3. Classifiers’ Selection

In the XGBIBS feature selection process of the XGB-Stacking method, different ensemble learning classifiers were chosen as the base classifiers, as these can effectively improve the accuracy of machine learning tasks. GBDT has outstanding performance in the field of prediction, and the improved methods based on GBDT include XGBoost, LightGBM, and CatBoost, all of which have their own advantages. GBDT, XGBoost, LightGBM, and CatBoost were chosen as the ensemble learning classifiers with AdaBoost and KNN to improve the difference of the classifiers. Therefore, six classifiers were selected in the experiment: KNN, AdaBoost, GBDT, XGBoost, LightGBM, and CatBoost.

In the model fusion by Sel-Stacking of the XGB-Stacking method, the base classifiers were selected from six classifiers after the XGBIBS feature selection, and the meta-classifier was SVM. SVM is more suitable for sample classification in a linear relationship because of the low model complexity, which can prevent overfitting.

### 4.2. Experimental Results of the XGBIBS Feature Selection Algorithm

In order to verify the superiority of the XGBIBS feature selection algorithm, comparative experiments were conducted from two perspectives: first, comparative experiments were done to prove the effectiveness of two XGBoost feature importance metrics and the BFGSBS strategy in the XGBIBS feature selection algorithm; secondly, this XGBIBS feature selection algorithm was compared with other feature selection algorithms to evaluate the overall performance of the algorithm.

In the experiments, the metrics of feature importance $I_{1}$ and $I_{2}$ were selected from Set I: gain, cover, weight, total_gain, total_cover. The experiment could not predict in advance which parameter could be used to obtain the best results from the different classifiers. Due to the limited number of indicators in Set I, the global optimum of the parameter combination could be obtained through enumeration.

The optimal feature subsets selected by different classifiers were different. The top ten features selected by XGBIBS algorithm were NEPHROPATHY, HEIGHT, HBA1C, CHD, LEADDP, OTHER_TUMOR, RESPIRATORY_SYSTEM_DISEASE, RENAL_FALIURE, HYPERLIPIDEMIA, and GYNECOLGICAL_TUMOR. Their scores and rankings are shown in Table 7.

#### 4.2.1. The Experimental Result of Using XGBoost Feature Importance Metrics

In order to verify the effectiveness of two XGBoost feature importance metrics in the XGBIBS algorithm, the search strategy was compared with the $I_{1}$-only ranking or the $I_{2}$-only ranking strategies. The comparison strategies were named BFGSBS1 and BFGSBS2, respectively. The figures from Table 8 give the experimental results of the classification accuracy. Table 8 also shows the classification effect of each classifier without feature selection (NoFS). Table 9 shows the influence of feature dimension reduction.

It can be seen from Table 8 that the two-metric strategy BFGSBS was obviously effective in the performance of most classifiers, compared with BFGSBS1 and BFGSBS2. The classification accuracy of BFGSBS1 and BFGSBS was the same for some classifiers, such as AdaBoost, XGBoost, and LightGBM. This was probably because BFGSBS included feature combinations in $I_{1}$, and no better solution was found with the BFGSBS strategy, which included most of the feature combinations in BFGSBS1. For all classifiers, BFGSBS achieved the highest classification effect. This shows that the two feature importance metrics could provide more feature combinations and avoid local optima.

It can be seen from Table 9 that compared with BFGSBS1 and BFGSBS2, BFGSBS performed better on four classifiers (KNN, AdaBoost, XGBoost, CatBoost). Therefore, the application of important metrics of different characteristics was helpful to improve DR. In general, there was little difference in the feature dimension reduction among the three strategies. Two feature importance metrics could improve the accuracy, but they would not weaken the feature dimension reduction.

#### 4.2.2. The Experimental Result of the BFGSBS Search Strategy

In order to verify the effectiveness of the BFGSBS strategy in the XGBIBS feature selection algorithm, BFGSBS was compared with the traditional Sequential Floating Backward Search (SFBS) strategy and Improved Sequential Floating Forward Search (ISFFS) [11]. The SFBS algorithm operates on one feature in the backward search process, and ISFFS is a forward search with different XGBoost feature importance metrics, which only operates on a single feature in a sequential floating forward search according to the effect of the evaluation function during iteration.

Table 10 shows the impact of different strategies on the classification accuracy. The BFGSBS strategy had the highest accuracy on the CatBoost classifier, which was 83.11%. The BFGSBS strategy had obvious advantages compared with SFBS, and it had more prominent performance on all classifiers, with the improvement of classification accuracy being between 0.66% and 4.01%. Compared with ISFFS, BFGSBS achieved relatively higher accuracy on the rest of the classifiers, except for KNN.

Table 11 shows the impact of different strategies on feature dimension reduction. BFGSBS had the highest feature dimension reduction on KNN, which was 82.35%. BFGSBS was significantly better than SFBS, while having a much lower effect than ISFFS. ISFFS had the highest feature dimension reduction on KNN, which was 88.24%. However, the high-dimensional reduction was at the cost of lower accuracy. Therefore, although BFGSBS used more features, the higher classification accuracy indicated that these features were not redundant features. BFGSBS fully exploited the joint advantages of multiple features.

#### 4.2.3. The Experimental Results of Different Feature Selection Algorithms

In order to verify the effectiveness of the XGBIBS method, comparison experiments with other feature selection algorithms, GA and SVM-RFE, were carried out. The experimental results are shown in Table 12 and Table 13.

It can be seen from Table 12 that the XGBIBS algorithm achieved the best results on most ensemble learning classifiers. Compared with the original classifiers without feature selection, the classification effect of XGBIBS was improved by 2.67–8.19%. Compared with SVM-RFE, XGBIBS had a higher or equal classification effect with respect to SVM-RFE. Compared with the genetic algorithm, except for the LightGBM classifier, the rest of the classifiers were better than the genetic algorithm. This shows that the XGBIBS algorithm had its advantages.

Analyzing the influence of different algorithms on the feature dimension reduction, XGBIBS had certain advantages in the improvement of feature dimension reduction on some classifiers. Although XGBIBS had a small feature dimension reduction on some classifiers, this did not impact the advantages of XGBIBS combined with the classification accuracy.

In order to compare the time cost of different feature selection algorithms, KNN was used as a classifier to calculate their time cost. Table 14 shows the runtime of different feature selection strategies and algorithms.

It can be seen from Table 15 that the runtime of the XGBIBS feature selection algorithm on the KNN classifier was 65.20 s, which was significantly higher than that of the algorithm without feature selection. The runtime of XGBIBS was similar to that of SVM-RFE, but much lower than that of GA. In general, although the runtime of the XGBIBS feature selection algorithm increased, the optimization of classification performance was obvious, so the time cost was acceptable.

In summary, XGBIBS had obvious advantages in small high-dimensional sample datasets at reducing redundant features, and the selected feature subsets had the best quality, which could improve the classification accuracy of the classifier.

### 4.3. The Experimental Results of Model Fusion by Sel-Stacking

Because the Sel-Stacking method improves the two aspects of the traditional model fusion method, experiments were performed from two aspects—the Label-Proba input strategy and the classifier combination strategy—to evaluate the performance of the method.

#### 4.3.1. The Experimental Results of the Meta-Classifier’s Different Input Strategies

This experiment verified the validity of the Label-Proba combination input. There were two comparison input strategies, the classification label input strategy and the classification Proba input strategy. The experimental results of the different input strategies are shown in Table 15.

The Label-Proba combined input with SVM as the meta-classifier had the best effect, which was 83.95%. In the model with SVM as the meta-classifier, the Label-Proba combination was 0.84% higher than the label strategy and 2.01% higher than the Proba strategy. To summarize the above experiments, it can be found that Label-Proba combined the input strategy could enhance the fitting ability of the stacking model fusion algorithm and achieve the best result.

#### 4.3.2. The Experimental Results of Different Classifier Combination Strategies

The experimental results showed that the base classifiers chosen by Sel-Stacking were KNN, GBDT, XGBoost, and CatBoost. These classifiers could achieve the highest classification accuracy of 83.95%.

In order to prove the effectiveness of the model fusion strategy of Sel-Stacking, this experiment compared this method with other model fusion methods and single classifiers.

The alternative classifiers were KNN, AdaBoost, GBDT, XGBoost, LightGBM, and CatBoost. The experiment also provided two ways to randomly select the combination of base classifiers in the stacking method to prove that the base classifiers selected by the Sel-Stacking method had better classification performance.

Other model fusion methods were as follows:

1. Stacking A: KNN, AdaBoost, GBDT, XGBoost, LightGBM, and CatBoost (all optional classifiers chosen as base classifiers in the stacking method);
2. Stacking B: AdaBoost, GBDT, XGBoost, LightGBM, and CatBoost (base classifiers Combination A by selecting randomly in the stacking method);
3. Stacking C: GBDT, XGBoost, LightGBM, and CatBoost (base classifiers Combination B by selecting randomly in the stacking method);
4. Voting A: KNN, AdaBoost, GBDT, XGBoost, LightGBM, and CatBoost (voting method using all optional classifiers);
5. Voting B: KNN, GBDT, XGBoost, and CatBoost (voting method using the base classifiers chosen by the Sel-Stacking method);
6. Blending A: KNN, AdaBoost, GBDT, XGBoost, LightGBM, and CatBoost (blending method using all optional classifiers);
7. Blending B: KNN, GBDT, XGBoost, and CatBoost (blending method using the base classifiers chosen by the Sel-Stacking method).

Single classifiers included all optional base classifiers (KNN, AdaBoost, GBDT, XGBoost, LightGBM, CatBoost) and other individual classifiers, such as SVM, LR, and random forest.

Table 16 shows the classification accuracy and runtime of different model fusion methods, and Table 17 shows the classification accuracy of the Sel-Stacking method and different single classifiers.

According to the experimental results in Table 16, the Sel-Stacking method through the global search reached the best accuracy of 83.95%. Compared with other model fusion methods, the proposed method of Sel-Stacking also had obvious advantages. It can be seen from the experimental results that if the model fusion method was not properly selected, the prediction accuracy was likely to be lower than that of a single classifier, such as Stacking B and Blending. However, it was obvious that the Sel-Stacking method could fully exploit the combined advantages of a single classifier.

Compared with other stacking model fusions, Sel-Stacking took about 1/3 more time to acquire the best performance. Although the time cost of this method was relatively high, in order to pursue higher accuracy, this runtime of tens of seconds was worth it.

A conclusion can be drawn for the experiments that the global optimal base classifier set was KNN, GBDT, XGBoost, and CatBoost, having the best effect. The combination of the above classifiers was the best because the base classifiers selected by this combination had good classification performance and had great differences in classifier construction.

It can be seen from Table 17 that the accuracy of the Sel-Stacking model was 0.84–9.7% higher than that of a single classifier. Compared with traditional machine learning classifiers and single ensemble learning classifiers, the Sel-Stacking model had the highest accuracy, which used the difference and diversity of single learners to make the results more robust and accurate.

## 5. Conclusions and Future Work

This paper proposed a model fusion algorithm, XGB-Stacking, based on XGBIBS feature selection and the Sel-stacking ensemble learning for the task of predicting diabetic retinopathy. The main aim of XGBIBS feature selection was to reduce the data feature redundancy and improve the effect of a single ensemble learning classifier. The buffer feature subset was added in the BFGSBS strategy to make it possible to operate on multiple features and make XGBIBS feature selection search for the optimal subset in different sequences based on different feature metrics of XGBoost. The Sel-Stacking model fusion method was used to solve the limitation of the generalization ability of a single classifier. In the Sel-Stacking model fusion method, the Label-Proba of the base classifiers was used as the input matrix of the meta-classifier, and the classifier combination method was searched globally to determine the optimal classifier combination. The method proposed in this paper was more suitable for the diabetic retinopathy dataset, and the accuracy of prediction on whether the patient had retinopathy was higher.

The aim of future studies will be to improve the feature dimensionality reduction rate of XGBIBS and to consider stacking model fusion methods combined with other algorithms to improve prediction accuracy.

## Figures and Tables

**Table 1:** All features of the data.

Feature Category	Number	Features
Basic Information	9	NATION, MARITAL_STATUS, SEX, AGE, BMI, BP_HIGH, BP_LOW, HEIGHT, WEIGHT
Disease Information	32	A_S, ARRHYTHMIAS, CHD, MI, LUNG_TUMOR, BILIARY_TRACT_DISEASE, CHF, CIRRHOSIS, BREAST_TUMOR, CAROTID_ARTERY_STENOSIS, CEREBRAL_APOPLEXTY, CLD, DIGESTIVE_CARCINOMA, ENDOCRINE_DISEASE, FLD, GYNECOLGICAL_TUMOR, HEMATONOSIS, HYPERLIPIDEMIA, LEADDP, HYPERTENTION, INTRACRANIAL_TUMOR, OTHER_TUMOR, MEN, NEPHROPATHY, PCOS, NERVOUS_SYSTEM_DISEASE, PREGNANT, PANCREATIC_DISEASE, RENAL_FALIURE, RESPIRATORY_SYSTEM_DISEASE, RHEUMATIC_IMMUNITY, UROLOGIC_NEOPLASMS
Biochemical Indicators	27	ALB, ALP, ALT, AST, DBILI, GGT, GLO, IBILI, LDH_L, TBILI, TP, BU, SCR, SUA, HDL_C, LDL_C, TC, TG, HB, PCV, PLT, GLU, HBA1C, APTT, FBG, PT, PTA

**Table 2:** Sex distribution of the samples.

SEX	Number of Samples	Percentage
Male (SEX = 1)	1869	62.51%
Female (SEX = 0)	1121	37.49%

**Table 3:** Age distribution of the samples.

AGE	Number of Samples	Percentage
Under 20 years old	2	0.07%
21–30	38	1.27%
31–40	139	4.65%
41–50	562	18.80%
51–60	1018	34.05%
61–70	860	28.76%
71 years old and above	371	12.41%

**Table 4:** Other basic qualitative information about the samples.

Features	Percentage (Value = 0)	Percentage (Value = 1)
NATION	95.65%	5.35%
MARITAL_STATUS	97.89%	2.11%

**Table 5:** Descriptions of the XGBoost feature importance measurement metrics.

Parameter	Description
weight	the number of times a feature is used to split the data across all trees
gain	the average gain of the feature when it is used in trees
cover	the average coverage of the feature when it is used in trees
total_gain	the total gain of the feature when it is used in trees
total_cover	the total coverage of the feature when it is used in trees

**Table 6:** The definition of the XGBIBS symbols.

Symbol	Definition
$I_{1}$	Queue arranged from small to large according to the importance of the XGBoost model $i_{1}$
$I_{2}$	Queue arranged from large to small according to the importance of the XGBoost model $i_{2}$
*O*	The buffer feature subset
Best_O	The optimal feature subset
Acc	Classification accuracy
Best_Acc	The highest classification accuracy
*N*	Traverse the control variables of the $I_{1}$ queue
*J*	Traverse the control variables of the $I_{2}$ queue

**Table 7:** Top 10 features of the XGB-Stacking model.

Feature	Ranking	Score
NEPHROPATHY	1	19.10
HEIGHT	2	4.95
HBA1C	3	3.69
CHD	4	3.57
LEADDP	5	3.40
OTHER_TUMOR	6	3.24
RESPIRATORY_SYSTEM_DISEASE	7	3.10
RENAL_FALIURE	8	2.69
HYPERLIPIDEMIA	9	2.48
GYNECOLGICAL_TUMORHEIGHT	10	2.25

**Table 8:** The influence of the XGBoost feature importance metrics on the classification accuracy.

Classifier	NoFS (%)	BFGSBS1 (%)	BFGSBS2 (%)	BFGSBS (%)
KNN	66.89	69.90	69.23	**75.08**
AdaBoost	78.09	**80.60**	80.43	**80.60**
GBDT	79.43	81.10	80.60	**82.27**
XGBoost	76.76	**82.27**	81.44	**82.27**
LightGBM	79.10	**81.77**	81.11	**81.77**
CatBoost	80.27	82.78	82.94	**83.11**

**Table 9:** The influence of the XGBoost feature importance metrics on DR.

Classifier	NoFS (%)	BFGSBS1 (%)	BFGSBS2 (%)	BFGSBS (%)
KNN	0	69.12	79.41	**82.35**
AdaBoost	0	**64.71**	47.06	**64.71**
GBDT	0	20.59	**54.41**	42.65
XGBoost	0	**42.65**	32.35	**42.65**
LightGBM	0	44.12	**47.06**	38.24
CatBoost	0	**51.47**	41.18	**51.47**

**Table 10:** Influence of different strategies on the classification accuracy.

Classifier	NoFS (%)	SFBS (%)	ISFFS (%)	BFGSBS (%)
KNN	66.89	71.07	**77.93**	75.08
AdaBoost	78.09	80.10	80.10	**80.60**
GBDT	79.43	81.61	76.92	**82.27**
XGBoost	76.76	80.77	77.93	**82.27**
LightGBM	79.10	81.10	75.08	**81.77**
CatBoost	80.27	82.44	77.42	**83.11**

**Table 11:** Influence of different strategies on DR.

Classifier	NoFS (%)	SFBS (%)	ISFFS (%)	BFGSBS (%)
KNN	0	25.00	**88.24**	82.35
AdaBoost	0	23.53	**79.41**	64.71
GBDT	0	22.06	**85.29**	42.65
XGBoost	0	20.59	**85.29**	42.65
LightGBM	0	19.12	**85.29**	38.24
CatBoost	0	82.35	**82.35**	51.47

**Table 12:** Performance of feature selection algorithms on classification accuracy.

Classifier	GA (%)	SVM-RFE (%)	XGBIBS (%)
KNN	73.24	72.07	**75.08**
AdaBoost	80.10	78.43	**80.60**
GBDT	81.77	80.94	**82.77**
XGBoost	81.27	81.94	**82.77**
LightGBM	**82.27**	81.10	81.77
CatBoost	81.77	**83.11**	**83.11**

**Table 13:** Performance of feature selection algorithms on DR.

Classifier	GA (%)	SVM-RFE (%)	XGBIBS (%)
KNN	55.88	33.82	**82.35**
AdaBoost	36.76	50.00	**64.71**
GBDT	36.76	**57.35**	42.65
XGBoost	44.12	**48.53**	42.65
LightGBM	51.47	**55.88**	38.24
CatBoost	47.06	22.06	**51.47**

**Table 14:** Runtime of different feature selection algorithms.

Feature Selection	Time (s)
NoFS	0.17
SVM-RFE	40.05
GA	827.28
BFGSBS	65.20

**Table 15:** Influence of the meta-classifier’s different input strategies on classification accuracy.

Input Strategy of the Meta-Classifier	Accuracy (%)
Label	83.11
Proba	81.94
Label-Proba	**83.95**

**Table 16:** The accuracy and running time of different model fusion methods.

Method	Accuracy (%)	Time (s)
Sel-Stacking	**83.95**	101.74
Stacking A	83.78	75.53
Stacking B	83.61	65.81
Stacking C	83.11	74.23
Voting A	83.44	8.79
Voting B	82.27	8.42
Blending A	80.10	8.49
Blending B	80.10	7.30

**Table 17:** The accuracy of the Sel-Stacking method and different single classifiers.

Algorithm Category	Algorithm	Accuracy (%)
model fusion method	Sel-Stacking	**83.95**
Single classifier	KNN	75.08
	AdaBoost	80.60
	GBDT	82.77
	XGBoost	82.27
	LightGBM	81.77
	CatBoost	83.11
	SVM	74.25
	LR	74.91
	Random Forest	76.58

## Data Availability

The dataset was from the General Hospital of Chinese people’s Liberation Army_ Diabetes complication early warning dataset. National population health science data center data warehouse, PhD, 2020 https://www.ncmi.cn//phda/dataDetails.do?id=CSTR:A0006.11.A0005.202006.001018 (accessed on 10 December 2020) Data license: (CCO) Public Domain Dedication.

## Abbreviations

The following abbreviations are used in this manuscript:

XGBIBS	Improved Backward Search Based on XGBoost
BFGSBS	Buffer Floating Generalized Sequential Backward Search
SFBS	Sequential Floating Backward Search
ISFFS	Improved Sequential Floating Forward Selection
